# Differential Interactions of Flavonoids with the Aryl Hydrocarbon Receptor In Silico and Their Impact on Receptor Activity In Vitro

**DOI:** 10.3390/ph17080980

**Published:** 2024-07-24

**Authors:** Monique Reis de Santana, Ylanna Bonfim dos Santos, Késsia Souza Santos, Manoelito Coelho Santos Junior, Mauricio Moraes Victor, Gabriel dos Santos Ramos, Ravena Pereira do Nascimento, Silvia Lima Costa

**Affiliations:** 1 Laboratory of Neurochemistry and Cellular Biology, Institute of Health Sciences, Federal University of Bahia, Salvador 40231-300, Brazil; monique.reis@ufba.br (M.R.d.S.); ravenanascimento@ufba.br (R.P.d.N.); 2 Molecular Modeling Laboratory, Department of Health, State University of Feira de Santana, Feira de Santana 44036-900, Brazil; ylanna.bonfim@gmail.com (Y.B.d.S.); kelsouzs14@gmail.com (K.S.S.); manoelito@uefs.br (M.C.S.J.); 3 Department of Organic Chemistry, Institute of Chemistry, Federal University of Bahia, Salvador 40231-300, Brazil; mmvictor@ufba.br (M.M.V.); gabrielramosquimica@gmail.com (G.d.S.R.); 4 National Institute of Translational Neuroscience (INNT), Rio de Janeiro 21941-902, Brazil

**Keywords:** AHR, cancer therapy, polyphenols, antagonist, TCDD, naringenin

## Abstract

The molecular mechanisms underlying the observed anticancer effects of flavonoids remain unclear. Increasing evidence shows that the aryl hydrocarbon receptor (AHR) plays a crucial role in neoplastic disease progression, establishing it as a potential drug target. This study evaluated the potential of hydroxy flavonoids, known for their anticancer properties, to interact with AHR, both in silico and in vitro, aiming to understand the mechanisms of action and identify selective AHR modulators. A PAS-B domain homology model was constructed to evaluate in silico interactions of chrysin, naringenin, quercetin apigenin and agathisflavone. The EROD activity assay measured the effects of flavonoids on AHR’s activity in human breast cancer cells (MCF7). Simulations showed that chrysin, apigenin, naringenin, and quercetin have the highest AHR binding affinity scores (−13.14 to −15.31), while agathisflavone showed low scores (−0.57 and −5.14). All tested flavonoids had the potential to inhibit AHR activity in a dose-dependent manner in the presence of an agonist (TCDD) in vitro. This study elucidates the distinct modulatory effects of flavonoids on AHR, emphasizing naringenin’s newly described antagonistic potential. It underscores the importance of understanding flavonoid’s molecular mechanisms, which is crucial for developing novel cancer therapies based on these molecules.

## 1. Introduction

The aryl hydrocarbon receptor (AHR) is an intracellular transcription factor that was first identified as important to binding exogenous ligands and mediating their toxic effects [1,2]. More recent studies emphasize its involvement in different cellular processes that include immune responses and cellular differentiation, evidencing its role as an important factor in neoplastic disease development and progression [3,4,5]. AHR sustained activity is observed in various tumor types and is often associated with unfavorable prognosis [1,6]. By regulating the expression of genes associated with cell differentiation, drug resistance, and inflammation induction, AHR’s activity has a significant impact on cancers such as human melanoma, breast cancer, liver cancer, lung cancer, and glioblastoma [6,7,8,9]. The abnormal AHR activity associated with the progression of cancer has indicated the receptor as a potential drug target for therapeutic applications [4].

Flavonoids, structure-diverse polyphenols found in plants, have been recognized for their well-established biological properties, including anticancer effects [10,11,12]. These natural compounds comprise a common structure of three rings, two of which are benzene rings interconnected by a heterocyclic ring. The classification of flavonoids is determined by their chemical structure, oxidation level, and substitution pattern of the heterocyclic pyran ring (C ring) [13]. Several flavonoids have been evaluated, in vitro and in vivo, for their pharmacological potential and their effect on the viability of human tumor cells, with various documented cellular effects, including differentiation, apoptosis, autophagy, cell cycle arrest, reactive oxygen species formation, metabolic modulation, and angiogenesis [14,15]. Studies have explored the pharmacological potential of flavonoids by investigating their cytotoxic activity and the potential mechanisms of response they mediate in tumor cells [16]. To date, among flavonoids with demonstrated anticancer activity, hydroxylated members stand out within their subclasses. As demonstrated in a previous study, the hydroxy flavonoids chrysin, naringenin, quercetin, apigenin, and agathisflavone (bis-apigenin) have been investigated for their efficacy in counteracting tumor growth [17]. We have shown that these flavonoids exhibit antiglioma effects in vitro and in vivo. The metabolic activity of flavonoids, chrysin, apigenin, rutin, and quercetin were investigated on human glioblastoma cell lines GL-15 and U251, demonstrating significant cytotoxicity, inhibited proliferation, and induced apoptosis in glioblastoma cells in a dose-dependent manner [18]. It has been demonstrated that apigenin inhibits proliferation, induces differentiation, and regulates the inflammatory profile of human and rat glioma cells [19]. Furthermore, rutin and its aglycone quercetin also exhibited antiglioma effects associated with the property of modulating the inflammatory profile of microglia. Moreover, the xenotransplantation of pretreated glioblastoma cells with the flavonoids rutin, quercetin, or apigenin into the brain of rats resulted in the loss of tumor neoformation competence [20]. Flavonoid naringenin was also studied for its antitumor effects. Research has shown that it inhibits cell cancer proliferation and/or migration and invasion [21]. Despite their well-documented anticancer properties, the precise mechanisms by which flavonoids exert their effects are yet not fully understood.

Natural products such as flavonoids are also AHR ligands [9]. Modeling studies show similarities and differences in the strength of interactions of flavonoids such as apigenin, naringenin, and quercetin, with AHR [22,23]. Nonetheless, some interactions have been contradictory and their structure-dependent binding patterns have to be further investigated. Furthermore, in vitro studies have demonstrated that flavonoids may exhibit specific agonist or antagonist effects depending on their structure and cell line specificity [22,23,24]. Screening studies of pharmaceutical-derived AHR modulators have been commonly conducted in breast cancer cell lines, such as MCF7 cells, due to their well-characterized AHR function and high responsiveness to its agonists [25]. These cells serve as a good model for investigating the expression of genes associated with AHR’s canonical pathway (CYP) [26]. This analysis consists of stimulating the AHR with a strong ligand such as 2,3,7,8-Tetrachlorodibenzo-p-dioxin (TCDD) and measuring the activity of its target genes, such as CYP1A1 (EROD activity), in fluorescence [27].

In this context, in this study, we aimed to deepen the understanding of the molecular mechanisms of flavonoids regarding their anticancer properties by evaluating the interaction patterns of the selected hydroxy flavonoids chrysin, naringenin, quercetin, apigenin, and agathisflavone within the AHR binding pocket; through molecular docking simulations; and also by investigating their potential as AHR antagonists in vitro. The results herein presented reiterate the potential of flavonoids to bind to AHR’s ligand pocket and reveal a dose-dependent antagonistic effect, which may be implicated in their exerted antitumor properties. Therefore, this work can contribute to a better understanding of how flavonoids modulate molecular targets such as AHR, potentially leading to significant advancements in drug development and their application in adjuvant tumor therapy.

## 2. Results

### 2.1. Homology Modeling and Model Validation of the Orthosteric Site (PAS-B) of AHR

**Figure 1:**
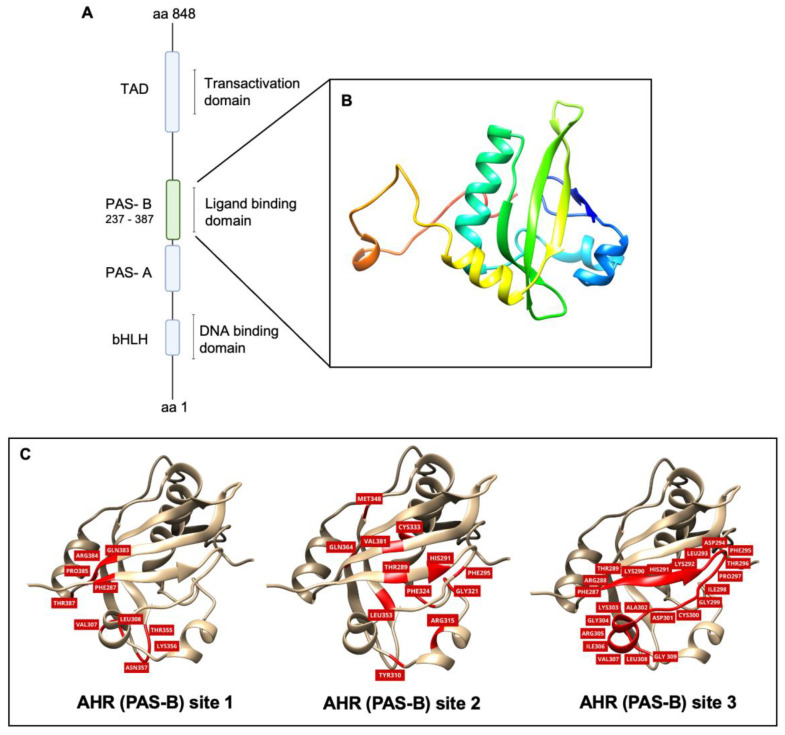
AHR PAS-B ligand-binding domain homology model. (**A**) Schematic structure of human AHR domains. Numbers at the domain boundaries refer to the amino acids of human proteins. (**B**) Three-dimensional representation of the PAS-B domain of human AHR. The model reveals the presence of four alpha helices along with a region characterized by antiparallel beta sheets. (**C**) Three-dimensional representation of 3 potential PAS-B ligand domain predicted binding sites. Amino acids of the binding pockets are highlighted in red. The binding regions were named based on which docking system they originated from. AHR PAS-B site 1: described in the work of Leclair et al., (2020) [23]; AHR PAS-B site 2: described in the work of SZÖLLÖSI et al. (2016) [28]; 3: calculated by CASTp (TIAN et al., 2018) [29] using the search radius of 1.4 Å. Three-dimensional models were built on the SWISS-MODEL platform.

Due to the absence of a complete structure of human AHRs, we employed a comparative modeling technique to generate a 3D model of the orthosteric site of AHR, specifically PAS-B, a sequence of 100 amino acids (residues 287–387) within the overall AHR sequence. Figure 1B shows the 3D model, with four alpha helices and an antiparallel beta sheets region. The Ramachandran plot evaluation indicated that 94.55% of residues are located in the most favored areas, 5.45% are located in permitted and generously allowed regions, and 0% are located in disallowed areas (Figure S1). The overlap of the model with the template (PDB ID 7VNA) was analyzed and it presented RMSD = 1.42 Å. After the molecular dynamic simulation, the RMSD analysis of the main chain was carried out to evaluate the flexibility of the protein globally and record deviations between the initial and final structure during a simulation. The results showed that the APO form stabilizes between 35 ns and 60 ns, that is, in this simulation time interval there are no conformational variations of a magnitude greater than 0.1 nm and, therefore, it was defined as the productive phase for the acquisition of data. The RMSF analyses were employed to assess the local fluctuation of each residue during the simulation. The two most prominent fluctuation peaks are observed between residues A373-G374 (exhibiting the highest fluctuation) and G309-A313.

### 2.2. Molecular Docking

Following the 3D model validation, three predicted binding sites for molecular recognition among ligands were selected, as outlined in the methodology (Figure 1C). The docking results are summarized in Table 1 Remarkable differences in binding affinity scores were evident between predicted sites 2 and 3 when compared to site 1, ranging from, −15.31 to −0.57, −15.27 to −5.14, and −5.46 to −0.94, respectively. The subsequent analysis was concentrated on binding sites 2 and 3. Upon fitting into the cavity of the AHR PAS-B model, each ligand exhibited comparable receptor binding affinity scores between sites 2 and 3, except for agathisflavone (−0.57 and −5.14). Chrysin (−15.14 and −15.27) and apigenin (−15.31 and −15.07) showed the top-ranked results for sites 2 and 3, respectively. Among the flavonoids evaluated, naringenin (−13.14 and −13.55) and quercetin (−13.52 and −13.59) showed intermediate FRED score values. Agathisflavone (−0.57 and −5.14) presented less affinity to the molecular target model.

**Figure 2:**
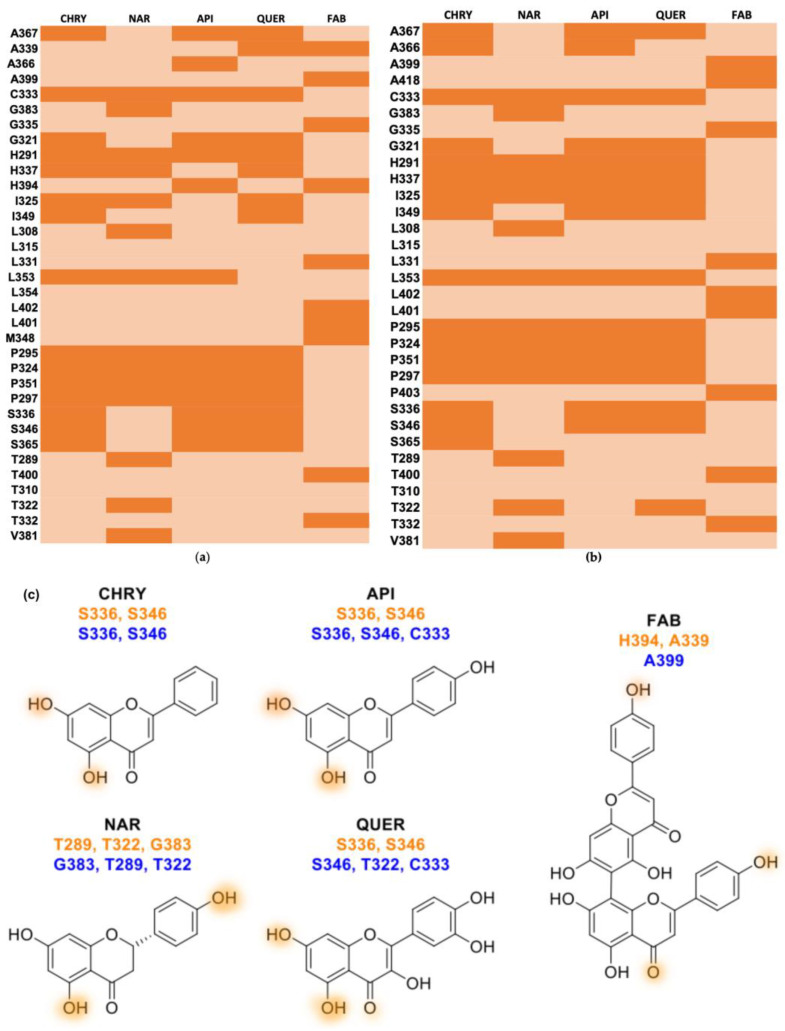
Flavonoids interact differently with the AHR binding domain (PAS-B). (**a**) Heat map showing the flavonoids chrysin (CHRY), apigenin (API), naringenin (NAR), quercetin (QUER), and agathisflavone (FAB) and their putative interactions with amino acids of the PAS-B domain (site 2). (**b**) Heat map showing the flavonoids CHRY, API, NAR, QUER, and FAB and their putative interactions with amino acids of the PAS- B domain (site 3). (**c**) Two-dimensional diagram of flavonoid structures and proposed hydroxyl group positions of CHRY, API, NAR, QUER, and FAB hydrogen bond interaction with AHR residues in the AHR binding site model (PAS-B). Orange-colored areas indicate hydrogen bonds with AHR residues at AHR binding sites (PAS-B). Residue names colored in orange indicate interactions with residues at model AHR binding site 2 (PAS-B), and names colored in blue indicate interactions with residues at AHR binding site 3 (PAS-B).

The prediction of amino acid interactions at binding sites 2 and 3 are shown in Figure 2 and Table 2. The simulations of flavonoids and AHR (PAS-B) binding site 2 (Figure 2a) showed broad hydrophobic interactions with residues C333, H291, H337, I325, L353, P295, P324, P351, and P297. Chrysin, apigenin, and quercetin formed donor hydrogen bonds with the hydroxyl groups of S336 and S346, whereas naringenin established these interactions with G383 (acceptor), T289, and T322 (donor). Hydrophobic interactions were also formed between flavonoids and AhR (PAS-B) binding site 3 with residues H291, H337, I325, L353, P295, P324, P351, and P297. However, apigenin–AhR and quercetin–AHR (PASB) binding at site 3 (Figure 2b) showed hydrogen bonds formed with C333. In the simulation of agathisflavone–AHR, hydrophobic contact was observed with A399, G335, L331, L402, L401, M348, T400, and T332 at AhR (PAS-B) binding site 2 and with A418, G335, L331, L402, L401, P403, T400, and T332 at AhR (PAS-B) binding site 3 (Table 2). Agathisflavone forms hydrogen bonds with H394 (acceptor) and A339 (donor) at binding site 2 and with A399 (acceptor) at binding site 3. These hydrogen bonds are indicated in orange-colored areas of the 2D flavonoid diagram in Figure 2c.

### 2.3. Flavonoids Inhibited AHR Activation In Vitro

Once we analyzed the potential of the flavonoids to bind AHR, we further investigated their ability to interact with and modulate the receptor’s activity in vitro. AHR activity was determined using the EROD activity assay, a standard method for evaluating the receptor’s canonical pathway by measuring CYP1A1 induction in MCF-7 human breast cancer cells. The MCF-7 cell line is a highly suitable model for studying AHR-mediated pathways due to its well-characterized AHR function and responsiveness to TCDD, making it ideal for evaluating the in vitro metabolism of flavonoids by CYP1A1 [27,30].

**Figure 3:**
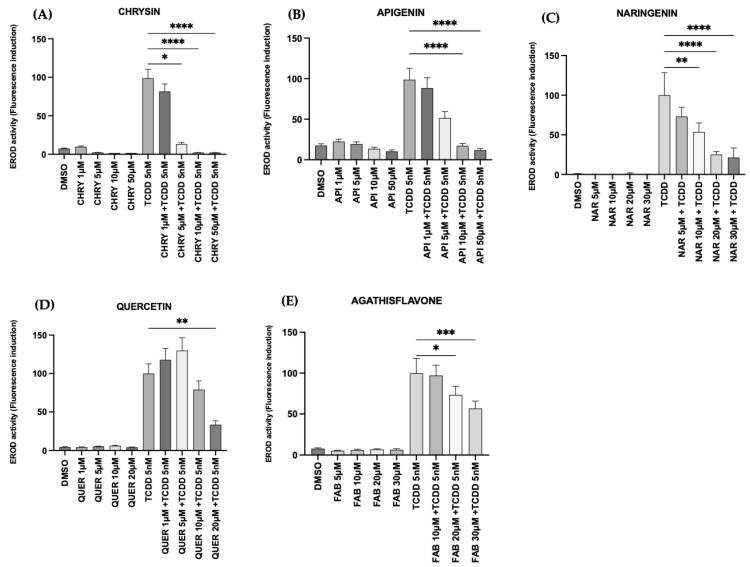
Flavonoids modulate the canonical AHR activity induced by TCDD. MCF7 cells were pretreated with the flavonoids (**A**) chrysin (1, 5, 10, 50 µM), (**B**) apigenin (1, 5, 10, 50 µM), (**C**) naringenin (5, 10, 20, 30 µM), (**D**) quercetin (1, 5, 10, 20 µM), and (**E**) agathisflavone (10, 20, 30 µM) for 2 h and exposed to agonist (TCDD 5 nM) for 6 h to measure the induction of CYP1A1 activity using an EROD activity assay. The CYP1A1 activity was analyzed considering the positive control condition (TCDD 5 nM). The results were compared to the control (100%) *n* = 3. The significance was evaluated by a one-way ANOVA test followed by the Tukey test; * *p* < 0.05, ** *p* < 0.01, *** *p* < 0.001, **** *p* < 0.0001.

All flavonoids exhibited significant in vitro CYP1A1 modulatory effects. At the lowest concentrations (1–10 µM), both flavonoids, chrysin and apigenin, reduced CYP1A1 activity in co-exposure in the presence of AHR’s agonist (TCDD 5 nM) (Figure 3A,B). Naringenin (10, 20, and 30 µM), quercetin (20 µM), and FAB (20 and 30 µM) pretreatments showed a concentration-dependent antagonistic activity in the presence of TCDD 5 nM (Figure 3C–E). As revealed by phase contrast microscopy, among the flavonoids tested chrysin and apigenin exhibited pronounced cytotoxicity at 50 µM, with cells presenting typical toxicity morphology within 2 h of exposure, compared to the control cultures exposed to DMSO (0.1%) (Figure S2).

## 3. Discussion

The AHR transcriptional activity regulates various cellular processes such as immune responses, cell cycle progression, proliferation, and cell differentiation [18]. Recent findings highlight a correlation between increased receptor activity and unfavorable prognosis in cancer [31]. In this study, we explored the potential of five hydroxy flavonoids, known for their anticancer properties, to bind to AHR and modulate its induced activity. Flavonoids are polyphenolic compounds widely distributed in the plant kingdom [32]. These molecules have been associated with several biological effects, including antioxidant and anti-inflammatory effects, cardiovascular protection, and potential anticancer properties [14,33]. Structural variations and cellular context contribute to the diverse effects of these compounds [34,35]. Our research aimed to contribute to understanding the molecular mechanisms of flavonoids and their potential interactions with AHR.

To gain a more comprehensive understanding of the AHR–ligand mechanism, a homology model of the AHR PAS-B region was constructed. Chrysin (CHRY), apigenin (API), naringenin (NAR), and quercetin (QUER) have similar basic structures, including a common flavonoid structure consisting of two benzene rings (rings A and B) connected by a three-carbon bridge (ring C), and their structures involve conjugated double bonds [13,16,36]. However, the types and positions of substituent groups (such as hydroxyl groups) attached to the structure differ. These differences influence the general three-dimensional arrangement of the molecules [37]. Variations in the arrangement and substitution of functional groups and the positioning of double bonds and global ring substitutions specifically contribute to differences in these compounds’ chemical and physical properties [38]. Notably, previous studies indicated the non-binding affinity of NAR to the PAS-B domain. However, in this study, the simulation of the NAR-AHR complex indicated robust interactions involving residues comparable to those that show strong interactions with CHRY, API, and QUER. This may be attributed to the assay systems used or the cellular context. Previous studies have indicated that the AHR PASB domain residues T289, H291, P295, P297, L308, L315, I325, P351, L353, A367, V381, and G383 are important to AHR-TCDD agonistic effects [28,36]. This indicates the potential interference of NAR in this interaction through hydrophobic contact with some of these residues (H291, P295, P297, L308, L315, I325, F351, L353, A367, V381) and the formation of a hydrogen bond with T289 and G388, thus supporting its antagonistic effect.

Although the presence of hydroxyl groups generally contributes to a better fit in the ligand pocket [22], the additional hydroxyl groups in agathisflavone (FAB) did not facilitate optimal fit to the binding sites identified in this study. From a structural point of view, FAB is a dimer of the flavonoid API, with each unit presenting a flavone structure comprising two benzene rings (rings A and B) and a three-carbon bridge (ring C) containing a double bond [23]. As a bioflavonoid, FAB has several hydroxyl groups in each flavone unit. A previous study suggested that ligand specificity may not be determined by PAS-B alone, and other proteins may influence ligand selection through protein-protein interactions that occur in the cytoplasmic complex [28]. This modulation may influence the activity of these factors and, consequently, the expression of their target genes.

In this study, we also investigated the potential of flavonoids to modulate the receptor’s activity by measuring the activity of an expressed gene associated with its canonical pathway (CYP). In this study, we investigated the ability of flavonoids to modulate CYP1A1 activity using an in vitro model (MCF7) mediated by AHR translocation and transcriptional activity. The MCF7 cell line is a highly suitable model for studying AHR-mediated pathways due to its well-characterized AHR function and high responsiveness to TCDD [39,40]. These cells express AHR, making them ideal for evaluating the in vitro metabolism of dietary flavonoids by CYP1A1 [41]. Previous studies have extensively used MCF7 cells to investigate AhR function and carcinogen activation [9,26,42,43]. Our findings showed that CHRY, API, NAR, QUER, and FAB have potent AHR antagonist effects and inhibited TCDD-induced CYP1A1 in MCF7 cells in a dose-dependent manner. These results were consistent with research studies that demonstrated the AHR-inhibition activity of flavonoids in different cell lines (Hepa-1c1c7, CaCO_2_, 501Mel, H4IIE) [21,44,45,46]. These molecules have been documented to influence the biology of several human cancers in vitro and in vivo, triggering apoptosis and inhibiting growth and migration [47]. Due to the high diversity and low toxicity of flavonoids, they have become an interesting candidate for research. For example, apigenin exhibits relevant antitumor activity. This compound can potentially induce the differentiation, apoptosis, and autophagy of glioma cells [19,48]. It can also modulate crucial signaling pathways involved in regulating immune responses, simultaneously inducing autophagy, and attenuating the survival, growth, proliferation, and migration of various types of cancer in vitro and in vivo [49]. In vitro studies suggest that CHRY and QUER exert anticancer effects on numerous types of cancer, including breast cancer, glioblastoma, liver cancer, pancreatic cancer, and lung cancer, by modulating different cellular processes [10,12,20,48]. These processes encompass angiogenesis, apoptosis, metastasis, autophagy, the cell cycle, and immune responses, achieved through activating or inhibiting distinct cell signaling pathways and molecules [50].

Among the five flavonoids tested, we specifically highlight the antagonistic effects demonstrated by NAR. This result is related to those observed in previous studies in CaCO_2_ and Hepa-1c1c7 cells, where NAR inhibited TCDD-induced CYP1A1 expression [22]. Recent studies suggest that NAR may have a potential effect on controlling the proliferation, invasion, and metastasis of different malignancies, such as colon cancer, lung neoplasms, breast cancer, leukemia and lymphoma, pancreatic cancer, liver cancer, brain tumors, melanoma, and cervical and ovarian cancer [21,44,45]. Considering its pharmacological profile, NAR can be exploited for therapeutic benefits [51,52]. FAB also reduced CYP1A1 activity levels in MCF7 cells. However, our docking results did not reveal a comparable binding score to the other flavonoids tested. The lack of significant binding interactions between FAB and AHR suggests that the positive impact of FAB on the inhibition of TCDD-induced CYP1A1 activity in MCF7 cells may not be attributed to a direct FAB-AHR interaction and requires further investigation. This study is a pioneer in exploring the impact of FAB on AHR and provides initial evidence of its ability to reduce CYP1A1 enzymatic activity. These findings indicate potential mechanisms related to the cytotoxic effects of FAB we have previously demonstrated in glioma cells (GL15 and U373) [53].

In summary, our findings confirm that hydroxy flavonoids can interact with the AHR PAS B domain. Also, they contribute to the knowledge of the potential of these molecules to modulate the receptor’s activity in an antagonistic way, in the presence of an agonist, and in vitro. These effects are structure- and dose-dependent. The present study highlights the promising potential of hydroxy flavonoids as effective modulators of AHR, particularly in the context of cancer therapy, and reveals NAR as a potential AHR antagonist.

## 4. Materials and Methods

### 4.1. Cell Culture

The human breast cancer cell line MCF7 was purchased from the European Collection of Cell Cultures (ECACC, Wiltshire, UK). The cells were propagated in humidified air (37 °C, 5% CO_2_) in 25 cm^2^ tissue culture vented cap flasks (Falcon^®^, Corning Inc., Saint-Quentin Fallavier, France, 353109) containing Dulbecco’s Modified Eagle Medium (DMEM) with 4500 mg/L D-glucose, 110 mg/L sodium pyruvate and non-essential amino acids, supplemented with 100 U/mL penicillin, 100 U/mL streptomycin, and 10% fetal bovine serum (FBS) (all from Gibco, ThermoFisher Scientific, Waltham, MA, USA). Upon reaching confluence, the medium was removed, and adherent cells were detached using trypsin solution (Gibco Trypsin-EDTA, ThermoFisher Scientific, Waltham, MA, USA) and seeded into 96-well Clear Flat Bottom plate (Falcon^®^, Corning Inc., Saint-Quentin Fallavier, France, 353072) at a density of 1 × 10^4^ cells/well.

### 4.2. Flavonoids and Treatments

Flavonoids were selected based on previous information on their potential anticancer effects [10,12] and their similarities in structure with a focus on the hydroxylated flavonoids chrysin (5,7-dihydroxyflavone), apigenin (4′,5,7-trihydroxy flavone), naringenin (4′,5,7-trihydroxy flavanone), and quercetin (3,3′,4′,5,6-pentahydroxyflavone) and the biflavonoid agathisflavone ((2S)-2-(3,4-Dihydroxyphenyl)-5,7-dihydroxy-3-(5,7-dihydroxy-4-oxo-4H-chromen-3-yl) chrome-4-one. The flavonoids chrysin (Aldrich, St. Louis, MO, USA 97% purity C80105) and quercetin (Aldrich, St. Louis, MO, USA, 95% purity, Q4951), specific for in vitro assays, were purchased commercially. Naringenin was obtained from the hydrolysis of naringin and (S)-naringenin. Apigenin was prepared from naringin [54], and agathisflavone was extracted from the aqueous extract of *P*. *pyramidalis* Tull leaves as previously described [55,56] (Table 3). All flavonoids were dissolved in dimethyl sulfoxide (DMSO; Sigma, Tokyo, Japan) to produce a 100 mM stock solution, which was stored and protected from light at −4 °C. DMSO was considered as control, a vehicle for diluting the molecules, in an equivalent volume (maximum 0.01%) and did not show a significant effect on the analyzed parameters when compared to cultures that were not exposed to this solvent. Ethoxyresorufin (EROD), salicylamide, and 2,3,7,8-tetrachlorodibenzo-p-dioxin (TCDD) were purchased from Sigma–Aldrich (St. Louis, MO, USA). The final dilutions of each of the molecules were prepared according to previous studies and, at the time of treatment, diluted directly in FBS-free DMEM [45,48,53].

### 4.3. Molecular Docking Study

#### 4.3.1. Homology Modeling

The protein sequence was obtained from UniProt (AHR in humans, code P35869). The primary sequence of the target protein was submitted to the SWISS-MODEL server for model construction. The templates and the target sequences were aligned using the tools available on the SWISS-MODEL server using the CLUSTAL OMEGA algorithm [57].

#### 4.3.2. Validation of the 3D Model of the AHR PAS-B Domain

The evaluation of the constructed model was carried out on the SWISS-MODEL server using the standard mean square deviation (RMSD) parameters, which evaluate the ability of the formed model to position itself correctly on the base mold; the evaluation was conducted by a RAMACHANDRAN graph [58]. Finally, the model was subjected to a molecular dynamics (MD) simulation to evaluate its stability. Molecular dynamics simulations were performed using the GROMACS package [59]. The correction for the protonation state of acidic and basic residues was adjusted in the pdb2gmx module of GROMACS 5.1.2 according to the optimum pH (7.0). The system was solvated using a dodecahedral box with the SPC-E water model [60]. The model was minimized using the initial stage’s Steepest Descent (SD) algorithm and then the Conjugated Gradient (GC) algorithm for 1000 cycles. After minimizing the energy of the system, the heating DM was carried out with a duration of 1ns in the isothermal–isobaric ensemble, NPT (number of particles, constant temperature, and pressure) (t = 100 ps), at a constant temperature of 300 K and restriction on the main chain of the protein; then, under the same conditions, a 100 ns production simulation was performed, without restriction on the main chain. The system’s stability was evaluated by analyzing the variation in the value of the root mean square deviation (RMSD) and root mean square fluctuation (RMSF) through the RMS and RMSF modules, respectively. These tools are available in GROMACS 5.1.2.

#### 4.3.3. Molecular Docking

Based on the literature on the orthosteric domain of PAS-B of AHR, three predicted binding sites were used for molecular recognition between ligands and the studied molecular target. Sites 1 and 2 were delimited based on previous studies [23,28]. Subsequently, the determination of site 3 was conducted through the application of CASTp methodology [29]. The flavonoids examined in this study are specified in Table 1. The molecular docking was performed using the FRED program of the OEDocking 4.2.0.2 package from OpenEye Scientific (OpenEye Scientific, Santa Fe, NM, USA) [61,62]. In the graphical utility Make Receptor 4.1.1.0 from OpenEye Scientific, the receiver was prepared by building a box for the coupling region using standardized program settings. The ligands were prepared by Omega 4.2.1.2 from OpenEye Scientific, which generates 3D conformations of the molecules subjected to the molecular coupling process, with 200 being the maximum number of conformers and a clustering threshold of 0.5 Å [63].

### 4.4. EROD Activity Assay

MCF7 cells were used as the gold standard to measure the induction activity of the cytochrome P4501A (CYP1A1) gene in vitro. For this, 200 µL of diluted cells (1 × 10^4^) was plated in each well of a 96-well Clear Flat Bottom plate (Falcon^®^, Corning Inc., Saint-Quentin Fallavier, France, 353072). After cells reached 100% confluency (48 h), cells were exposed to flavonoids at 1–50 µM for 2 h. Then, cells were exposed to 5 nM of the AHR agonist 2,3,7,8-tetrachlorodibenzo-p-dioxin (Sigma-Aldrich, Saint-Quentin Fallavier, France), pretreated or not with flavonoids, for 6 h. After the exposure time, the medium was removed and the wells were rinsed with 200 μL of PBS immediately. Then, 100 μL of phosphate-buffered saline pH 7.4 containing 50 μM ethoxy resorufin from Sigma–Aldrich (St. Louis, MO, USA) and 1.5 mM of salicylamide from Sigma–Aldrich (St. Louis, MO, USA) were added to each well and the plate was incubated in a humidified chamber with 5% CO_2_ at 37 °C for 20 min. Fluorescence was measured at an excitation wavelength of 535 nm and an emission wavelength of 590 nm with a SpectraMax Gemini XS spectrofluorometer (Molecular Devices, San Jose, CA, USA) carried out at 37 °C for 30 min [27,30,64].

### 4.5. Statistical Analysis

Data were statistically analyzed using GraphPad Prism 9 software (GraphPad, San Diego, CA, USA) for Mac. Experimental results are presented as means ± standard deviation (SD). Variance analysis was performed using a one-way ANOVA test, followed by the Tukey post-test. Parametric statistical tests were employed for comparisons between treatment groups and control groups. Statistical differences were considered significant at *p* ≤ 0.05. All experiments were repeated at least three times.

## 5. Conclusions

The results of this study affirm the significant potential of flavonoids in modulating AHR. Molecular docking revealed that flavonoids—chrysin, apigenin, naringenin, and quercetin—interact with a common set of AHR residues within the AHR PAS-B binding site, displaying the potential to interact with similar residues required for agonists. This potential is confirmed in this research, in which flavonoids inhibited AHR’s activity in the presence of TCDD in vitro. These findings reveal a potential molecular mechanism involved in the anticancer properties of flavonoids and contribute to future investigations in exploring novel therapeutic perspectives for neoplastic diseases, especially regarding AHR’s activity modulation.

## Figures and Tables

**Table 1:** Binding energy values of potential AHR ligands in predicted AHR (PAS-B) binding sites.

Flavonoid	AHR (PAS-B) Site 1	FRED Chemgauss4 Score AHR (PAS-B) Site 2	AHR (PAS-B) Site 3
Chrysin	−4.93	−15.14	−15.27
Apigenin	−4.73	−15.31	−15.07
Naringenin	−5.46	−13.14	−13.55
Quercetin	−4.51	−13.62	−13.59
Agathisflavone	−0.94	−0.57	−5.14

**Table 2:** Amino acid interactions between flavonoids and AHR (PAS-B) predicted sites (2 and 3).

	Flavonoid	Hydrophobic Contact	Hydrogen Bond Aceptor	Hydrogen Bond Donor
AHR (PAS-B) site 2	Chrysin	A367 C333 G321 H291 H337 I325 I349 L353 P295 P324 P351 P297 S365	−	S336 S346
	Apigenin	A367 A366 C333 G321 H291 H337 I325 I349 L353 P295 P324 P351 P297 S365	−	S336 S346
	Naringenin	C333 H291 H337 I325 L308 L353 P295 P324 P351 P297 V381	G383	T289 T322
	Quercetin	A367 A366 C333 G321 H291 H337 I325 I349 P295 P324 P351 P297 S365	−	S336 S346
	Agathisflavone	A399 G335 L331 L402 L401 M348 T400 T332	H394	A339
AHR (PAS-B) site 3	Chrysin	A367 A366 C333 G321 H291 H337 I325 I349 L353 P295 P324 P351 P297 S365	−	S336 S346
	Apigenin	A367 A366 G321 H291 H337 I325 I349 L353 P295 P324 P351 P297	C333	S336 S346
	Naringenin	C333 H291 H337 I325 L308 L353 P295 P324 P351 P297 V381	G383	T289 T322
	Quercetin	A367 G321 H291 H337 I325 I349 L353 P295 P324 P351 P297 S336	C333	S336 S346
	Agathisflavone	A418 G335 L331 L402 L401 P403 T400 T332	A399	−

**Table 3:** Flavonoids’ schematic structure. Models generated by MolView 2.4.

Flavonoid	Empirical Formula (Hill Notation)	Schematic Structure
Chrysin	C_15_H_10_O_4_	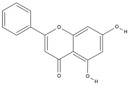
Apigenin	C_15_H_10_O_5_	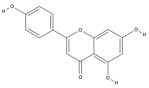
Naringenin	C_15_H_12_O_5_	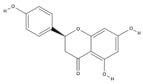
Quercetin	C_15_H_10_O_7_	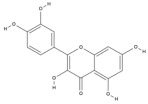
Agathisflavone	C_30_H_18_O_10_	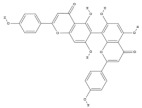

## Data Availability

The original data presented in the study are included in the article; further inquiries can be directed to the corresponding author.

## Supplementary Materials

The following supporting information can be downloaded at https://www.mdpi.com/article/10.3390/ph17080980/s1, Figure S1: Ramachandran plot. Figure S2: Cytotoxicity of flavonoids chrysin and apigenin in MCF7 cells.
