# Supplementary material for: Differential Interactions of Flavonoids with the Aryl Hydrocarbon Receptor In Silico and Their Impact on Receptor Activity In Vitro
Source: Pharmaceuticals (Basel). 2024 Jul 24;17(8):980. doi: 10.3390/ph17080980 (PMC11356971; doi:10.3390/ph17080980)
Supplement: Supplementary file 1 [file pharmaceuticals-17-00980-s001.zip › pharmaceuticals-3090757-supplementary.pdf]

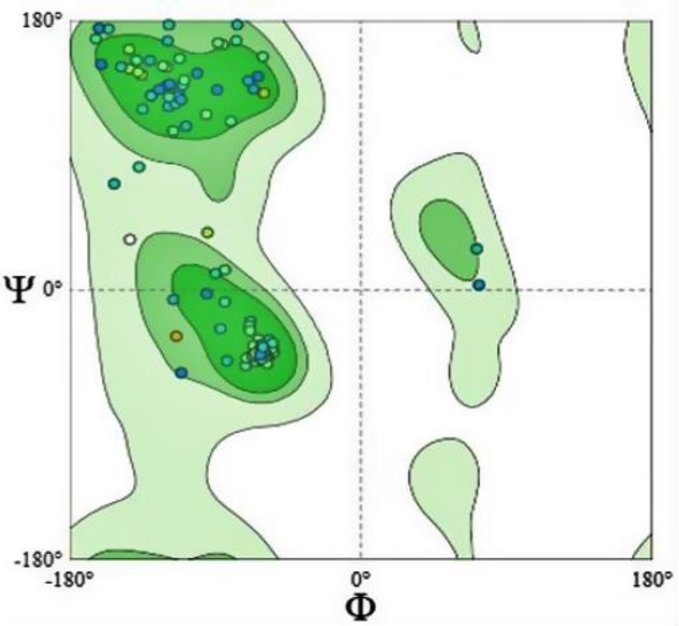

Figure S1. Ramachandran chart representation. Amino acids (spheres), most favorable regions (dark green), permitted regions (medium green), generously permitted regions (light green), and not allowed (white). Graphic generated on the SWISSMODEL server.

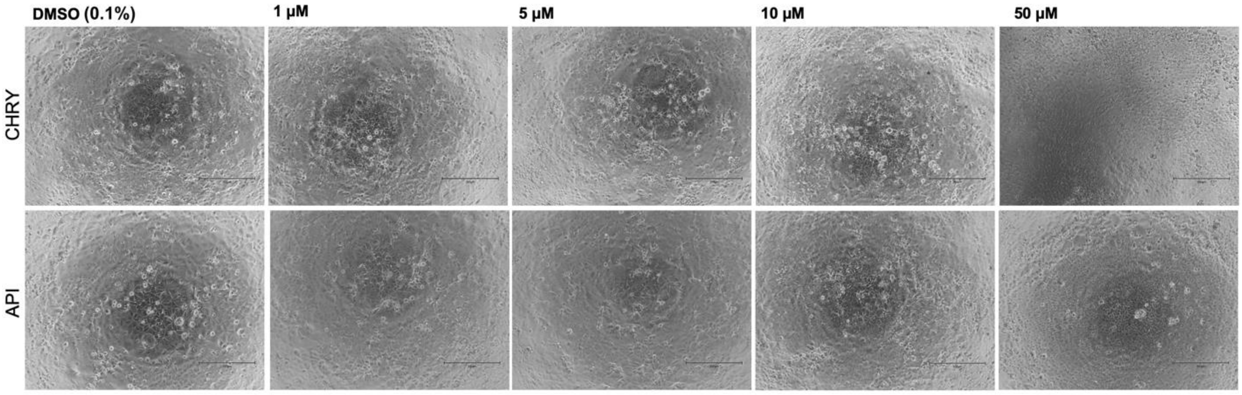

Figure S2. Effects of chrysin (CHRY) and apigenin (API) on the viability of human MCF7 cells. Phase contrast photomicrographs MCF7cell cultures exposed to CHRY and API (1, 5, 10 and 50 μM) and DMSO control condition (0.01%), after 2 h; scale bar = 300 μm
